# Insulin Clearance Along the Liver–Kidney Axis: Implications for Insulin Action

**DOI:** 10.3390/metabo16070439

**Published:** 2026-06-24

**Authors:** Germán Perdomo, Irene Cózar-Castellano, Sonia M. Najjar

**Affiliations:** 1 Instituto de Biomedicina y Genética Molecular (Consejo Superior de Investigaciones Científicas-Universidad de Valladolid), 47003 Valladolid, Spain; irene.cozar@uva.es; 2 Instituto Biosanitario de Valladolid (IBioVALL), 47003 Valladolid, Spain; 3 Centro de Investigación Biomédica en Red de Diabetes y Enfermedades Metabólicas Asociadas (CIBERDEM), 28029 Madrid, Spain; 4 Department of Biomedical Sciences, Heritage College of Osteopathic Medicine, Ohio University, Athens, OH 45701, USA; 5 Diabetes Institute, Heritage College of Osteopathic Medicine, Ohio University, Athens, OH 45701, USA

**Keywords:** insulin clearance, insulin endocytosis, insulin degradation, CEACAM1, IDE, LRP1, LRP2

## Abstract

The pleiotropic actions of insulin are mediated by cascades of signaling pathways and are regulated by circulating insulin levels. Under physiologic conditions, insulin levels reflect the balance between pancreatic beta-cell secretion and insulin clearance, which occurs primarily in liver hepatocytes and, to a lesser extent, in kidney proximal tubule cells. Therefore, coordination between insulin secretion and clearance is essential for systemic insulin sensitivity. Whereas insulin secretion is widely investigated, exploring the role of insulin clearance in regulating insulin sensitivity remains limited. This review summarizes the main mechanisms underlying insulin clearance along the liver–kidney axis and discusses how they contribute to metabolic regulation in health and disease.

## 1. Introduction

Insulin is a key hormone that governs glucose and lipid homeostasis. Dysregulation of its circulating levels leads to altered metabolism [1,2,3].

Circulating insulin levels is determined not only by its secretion from pancreatic β-cells but also by its extraction from the blood [4]. The liver–kidney axis establishes a portal-to-systemic gradient that regulates peripheral insulin levels and fine-tunes insulin secretion from pancreatic β-cells for optimal metabolic control. Hepatic first-pass extraction (~50–80%), renal clearance (~20–40%) and removal by other tissues, such as skeletal muscle, adipose tissue, and the brain, collectively calibrate circulating insulin levels to potentially exert a feedback effect on pancreatic β-cell secretory function [5,6,7]. Circulating insulin has a short half-life of 4–6 min and is cleared at a rate of 320–400 mL/min in liver and 200 mL/min in kidney. This highlights the dynamic nature of insulin’s turnover [5].

The causal relationship between impaired insulin clearance and insulin resistance remains a “chicken-and-egg” open question. On the one hand, hyperinsulinemia may result from compensatory insulin hypersecretion and reduced insulin clearance to offset systemic insulin resistance commonly associated with excessive abdominal obesity [8,9]. In this context, impaired insulin clearance serves to prolong insulin’s half-life and diminish the burden on pancreatic beta cells [9]. On the other hand, reduced insulin clearance causes chronic hyperinsulinemia and subsequently, insulin resistance [9,10]. Thus, depending on the physiologic framework, impaired insulin clearance may arise secondarily to systemic insulin resistance or play a primary role in initiating hyperinsulinemia-driven insulin resistance [2,11].

Impaired hepatic insulin clearance causes chronic hyperinsulinemia, which promotes hepatic insulin resistance at least in part by downregulating insulin receptors (INSR) [8,12,13]. This process also favors de novo lipogenesis, with subsequent lipid redistribution to adipose tissue for storage. If these changes are sustained, they may stimulate adipokine release and enhance lipolysis-derived fatty acid flux. Together, these processes contribute to systemic insulin resistance, independently of insulin secretion [14].

There is growing evidence that impaired insulin clearance contributes significantly to the risk of metabolic syndrome, particularly in Native American, African American, and Hispanic populations [15,16,17,18]. Moreover, the Yale Pediatric Cohort study demonstrated a strong correlation between intrahepatic lipid accumulation with reduced endogenous insulin clearance and hepatic insulin resistance in obese youths irrespective of ethnicity [19]. Additionally, increased liver lipid content and glycemic dysregulation appear to relate more differentially to reduced insulin clearance in recent-onset type 2 and type 1 diabetes [20]. Therefore, it is imperative to delineate the molecular and physiological mechanisms underlying dysregulated insulin clearance and to define its role in the pathogenesis of insulin resistance and related metabolic disorders.

## 2. Hepatic Insulin Clearance

Insulin is secreted from pancreatic β-cells in discrete pulses approximately every 4–5 min. These insulin pulses reach the liver initially via the portal vein through capillary fenestrae [21,22]. In healthy humans, hepatocytes preferentially remove up to ~80% of insulin secreted during these bursts as compared to in between peaks [23]. This mechanism effectively reduces the high amplitude of portal vein oscillations by ~100-fold, creating a stable and uniform insulin concentration in systemic arterial blood [22]. This secures appropriate levels of insulin reaching extrahepatic peripheral tissues to trigger proper systemic insulin action [24,25,26,27]. In this manner, the mechanism of pulse-dependent gating governs both hepatic insulin exposure and peripheral delivery, thereby establishing a pancreas–liver feedback axis that is indispensable for metabolic regulation [22]. In early type 2 diabetes (T2D) and obesity, reduced pulse mass (amplitude) impairs clearance efficiency and elevates circulating insulin levels [28,29].

Hepatocytes, the most dominant cell population in liver, are the major sites of endogenous insulin clearance. Binding of acutely released insulin to its receptor on the sinusoidal domain of hepatocytes, activates its tyrosine kinase activity and leads to its autophosphorylation as well as the phosphorylation of several other substrates. It also triggers receptor-mediated insulin endocytosis to target insulin to its degradation process via clathrin-coated pits and vesicles. Receptor-mediated insulin endocytosis and degradation constitute the basic mechanisms of insulin clearance in hepatocytes [8].

In addition to INSR, several chaperone proteins, such as carcinoembryonic antigen-related cell adhesion molecule 1 (CEACAM1), together with receptors like low-density lipoprotein receptor-related protein 1 (LRP1), and proteolytic enzymes, including Insulin-Degrading Enzyme (IDE), mediate receptor-dependent insulin endocytosis and clearance in the liver [8,30,31,32]. Below, we present the experimental evidence supporting the role of each factor in insulin extraction from the bloodstream and their implication in metabolic regulation.

### 2.1. Role of CEACAM1 in Receptor-Mediated Insulin Uptake in Hepatocytes

By comparison to other INSR substrates, CEACAM1 is uniquely poised to play a role in hepatic insulin clearance. First, it is a plasma membrane glycoprotein of the sinusoidal domain of hepatocytes. Second, it is largely expressed in major sites of insulin clearance (liver and kidney), but not in skeletal muscle or adipose tissue, which play a relatively marginal role in insulin clearance [8].

**Figure 1:**
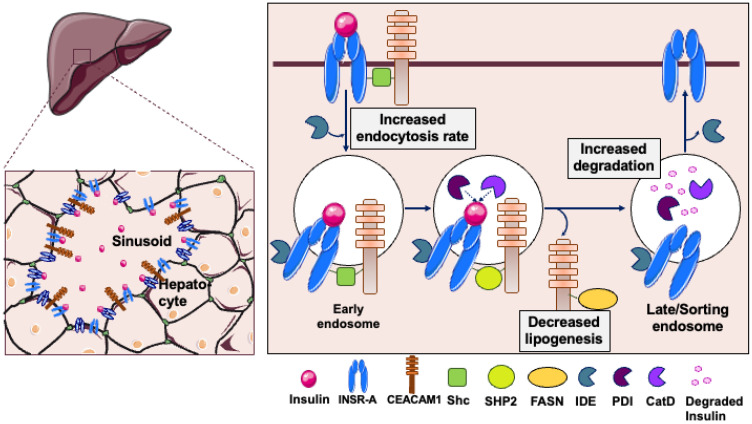
Schematic diagram summarizing enhanced receptor-mediated insulin endocytosis in hepatocytes by CEACAM1. Upon its phosphorylation by the insulin receptor (INSR), CEACAM1 partakes in the insulin–INSR complex to stabilize it and increase its rate of uptake followed by its degradation. The latter step is facilitated by FASN binding to CEACAM1 to pull it off the complex. This leads to suppression of FASN activity in response to an acute rise of insulin in the portal circulation.

CEACAM1 is alternatively spliced into two isoforms differing by the length of their intracellular tails and containing four IgG-like structures in their extracellular domains. The 71-amino-acid intracellular tail of the long isoform (CEACAM1-4L) includes tyrosine phosphorylation sites that are lacking in the 11-amino-acid tail of the short isoform (CEACAM1-4S) [33]. Whereas CEACAM1-4S is exclusively expressed on the bile canalicular domain of hepatocytes, CEACAM1-4L is expressed in the sinusoidal domain where it promotes receptor-mediated insulin uptake in response to pulses of secretory insulin [8,34,35]. Upon its phosphorylation on Tyr488 by INSR, CEACAM1-4L (hereafter termed CEACAM1 for simplicity) recruits Shc from the perinuclear region to bind to Tyr960 in INSR via its PTP binding domain [8] (Figure 1). CEACAM1 binding stabilizes the insulin-INSR complex and enhances its rate of endocytosis into clathrin-coated pits [8]. Ultimately, it recruits SHP2 instead of Shc to block PI3Kinase, while targeting the complex to late endosomes for degradation [8].

In this manner, CEACAM1 partakes in the insulin–INSR endocytosis complex primarily through the low-abundant INSR-A isoform, which binds to insulin with higher affinity than the more prevalent INSR-B isoform [36]. In fact, the dual-receptor system of INSR-A and INSR-B coordinates the regulation of insulin clearance [37]. At low physiologic insulin levels, INSR-A efficiently captures and internalizes insulin due to its low *Kd* equilibrium dissociation constant. As insulin levels rise, the more abundant INSR-B mediates bulk clearance to ensure linear uptake and prevent receptor saturation. Combined, this produces a concentration-dependent clearance curve that preserves insulin homeostasis.

From an evolutionary perspective, such a dual-receptor system likely emerged as a robust adaptive response to changes in energy availability [38,39]. High-affinity INSR-A receptors evolved first to detect trace levels of insulin and enable survival signaling when ancestral nutrients were scarce. With the emergence of energy resources, the abundant low-affinity INSR-B evolved to efficiently handle postprandial hormone surges, thereby averting the toxicity of excess insulin and limiting insulin resistance.

Moreover, it has been suggested that the differential binding affinity of INSR-A and INSR-B may render hepatic insulin receptors less sensitive to hyperinsulinemia-induced receptor downregulation, thereby protecting the liver against the two- to three-fold higher portal insulin concentrations relative to peripheral circulation [40]. Thus, by promoting insulin-induced INSR-A internalization, CEACAM1 may regulate the relative distribution of INSR-A and INSR-B in the hepatocyte, consistent with its important role in regulating hepatic insulin sensitivity.

Nevertheless, following its internalization, insulin dissociates from its receptor in late endosomes to undergo degradation in the acidic milieu of late endosomes, while INSR recycles back to the plasma membrane [8]. The destabilization of the complex is achieved by the binding of fatty acid synthase (FASN), predominantly distributed in the perinuclear region of hepatocytes, to Tyr488 of CEACAM1 [41].

Under normal physiologic conditions, the binding of tyrosyl-phosphorylated CEACAM1 in response to insulin pulses suppresses the enzymatic activity of FASN [41]. This acutely inhibits de novo lipogenesis, thereby protecting the liver from the lipogenic effect of high portal insulin levels. However, in chronic hyperinsulinemia, as occurs in early T2D and obesity, the pulsatility of insulin release is lost [1,21,28,42,43], leading to reduced INSR activation and subsequently, hepatic insulin resistance [14,44] in addition to increased de novo lipogenesis (hepatic steatosis) and gluconeogenesis [1,45].

This unifying mechanism—linking the pulsatility of insulin secretion and clearance on one hand to the acute suppression of de novo lipogenesis and the maintenance of hepatic insulin sensitivity on the other—challenges the prevailing concept of selective hepatic insulin resistance. This paradoxical selectivity state is characterized by impaired insulin-mediated suppression of hepatic gluconeogenesis, while lipogenesis is increased to drive fatty liver disease in the presence of systemic hyperinsulinemia. It has been widely accepted that insulin signaling decouples to impair suppression of gluconeogenesis (via defective PI3K-Akt-FOXO1) [27,46,47] while hyperactivating lipogenesis (via preserved transcriptional activation of SREBP-1c/ChREBP) [48]. However, diminished CEACAM1 phosphorylation disrupts both insulin clearance and acute FASN inhibition via a common proximal defect, resulting in hyperglycemia, hepatic steatosis and hepatic insulin resistance. This is supported by clinical studies failing to show selective hepatic insulin clearance in patients with metabolic dysfunction-associated steatotic liver disease (MASLD) [49] and attributing chronic hyperinsulinemia in these patients to impaired insulin clearance [50]. Thus, CEACAM1 phosphorylation by INSR links signaling triggered by secretory insulin bursts to insulin clearance and its suppression of lipogenesis as well as gluconeogenesis [8,51].

Global *Ceacam1* knockout (*Cc1*^−/−^) mice develop chronic hyperinsulinemia, primarily due to impaired insulin clearance [52] (Table S1). This is accompanied by systemic insulin resistance, hepatic steatosis owing to induced de novo lipogenesis and reduced fatty acid β-oxidation (FAO), visceral obesity (owing in part to hyperphagia) [53], and markedly accelerated progression to MASH (including hepatic fibrosis) when challenged with a high-fat diet [54]. Additionally, these mice develop endothelial dysfunction with increased leukocyte adhesion to vascular walls, cardiac hypertrophy, and spontaneous hypertension [55,56,57]. Consistent with the key role that CEACAM1 plays in metabolic control, transgenic liver-specific reconstitution of CEACAM1-4L dramatically reverses the entire metabolic syndrome in global *Cc1*^−/−^ null mice, beginning with normalization of insulin clearance [54]. Similarly, acute adenoviral-dependent liver delivery of wild-type but not of the phosphorylation-defective Serine-503-Alanine mutant of CEACAM1-4L rapidly prevents chronic hyperinsulinemia, insulin resistance, and steatohepatitis caused by feeding C57BL6/J mice a high-fat diet [58].

Liver-specific *Cc1* mutants [14,59] and transgenic L-SACC1 mice [44] with liver-specific inactivation of CEACAM1 overexpressing a dominant-negative non-phosphorylatable serine503-to-alanine mutant of CEACAM1-4L recapitulate the metabolic phenotype of *Cc1*^−/−^ nulls. The phenotypes of these mice show that defective hepatic insulin clearance drives hyperinsulinemia, which in turn, causes systemic insulin resistance, increased visceral adiposity and MASH-like pathology characterized by steatosis (upregulation of de novo lipogenesis through SREBP-1c transcriptional activation of lipogenic genes), inflammation and hepatic fibrosis, particularly evident under high-fat feeding [54,60]. Furthermore, liver-specific *Ceacam1* mutants develop atherosclerosis when propagated on the *Ldlr* null background and fed with an atherogenic diet [61], further demonstrating that the loss of CEACAM1 in hepatocytes does not only cause MASH, but bridges it to atherosclerosis.

In contrast, liver-specific transgenic overexpression of wild-type CEACAM1-4L (L-CC1) prophylactically prevents defects in insulin clearance, even under metabolic stress [62]. It also markedly protects systemic insulin sensitivity against prolonged high-fat intake in addition to suppressing its resultant hepatic steatosis, dyslipidemia, and adipose tissue fibrosis [62]. Recently, we have shown that liver-specific transgenic overexpression of wild-type CEACAM1-4L prevents age-related insulin resistance, steatohepatitis and hepatic fibrosis, in addition to conferring survival advantage [63].

Collectively, these complementary genetic models demonstrate that CEACAM1 deficiency predominantly initiates systemic cardiometabolic dysfunction through liver autonomous defects in insulin signaling and clearance. Moreover, they confirm the proximal molecular requirement for CEACAM1 tyrosine phosphorylation in integrating insulin signaling with cardiometabolic homeostasis.

This compelling CEACAM1-related preclinical evidence supports the alternative hypothesis in which impaired hepatic insulin extraction drives systemic insulin resistance, rather than merely contributing to compensatory hyperinsulinemia secondary to increased insulin secretion [2,4,8,9,51]. Consistently, hyperinsulinemia in patients with MASLD has been attributed primarily to defective insulin clearance rather than its overproduction [10,16,50,64]. Further reinforcing the central role of hepatic CEACAM1, its expression has been found to be significantly reduced in obese rodents and humans with insulin resistance and MASLD independent of T2D [65,66,67], as well as in patients with metabolic dysfunction-associated steatohepatitis (MASH) [59]. Moreover, circulating soluble CEACAM1 levels correlate inversely with insulin clearance in patients exhibiting insulin resistance, glucose intolerance, and elevated fatty liver index, as observed in the Portuguese PREVADIAB2 study [68].

### 2.2. Role of LRP1 in Insulin Uptake in Hepatocytes

Low-Density Lipoprotein Receptor-Related Protein 1 (LRP1) is highly expressed in liver (and to some extent in kidney), among other tissues [69,70,71,72]. As a receptor for chylomicron remnants in liver, LRP1 clears ApoE-rich lipoproteins, contributes to cholesterol homeostasis and supports hepatic lipid turnover and energy balance [73].

LRP1 is produced as a ~600 kDa precursor that is processed into a large extracellular α-chain and an ~85 kDa β-chain that crosses the plasma membrane [69,74]. It undergoes rapid recycling between perinuclear endosomal compartments and the plasma membrane, preferentially the basolateral domain [75]. This is guided by the cytosolic NPxY and dileucine motifs that recruit clathrin/AP-2 and adaptor proteins such as Dab1/2 [76,77,78,79].

In hepatocytes, insulin promotes LRP1 translocation to the plasma membrane through the INSR/PI3K/Akt/GSK3β signaling pathway to enhance insulin response and facilitate glycogen synthesis [80,81]. Hepatocyte-specific deletion of *Lrp1* gene causes hepatic insulin resistance, steatosis, and dysregulated fatty acid metabolism, particularly under high-fat feeding conditions [74]. Whereas LRP1 regulates intracellular trafficking [76], it does not directly mediate insulin endocytosis. Instead, it contributes to receptor-mediated insulin endocytosis in hepatocytes by regulating INSR abundance and plasma membrane lipid composition [81].

### 2.3. Role of Insulin-Degrading Enzyme in Insulin Clearance in Hepatocytes

Insulin-degrading enzyme (IDE), a zinc metalloprotease, is predominantly expressed in liver. In hepatocytes, IDE exists primarily as a single full-length 1019-amino-acid protein (~110 kDa). Post-translational modifications and tetramerization under oxidizing conditions regulate its enzymatic activity [82].

IDE exists as two distinct variants that are compartmentalized to the cytosol (cIDE) or to the mitochondria (mIDE; inner mitochondrial membrane and matrix). Whereas mIDE is potentially involved in modulating organelle-specific metabolism, cIDE is mostly involved in peptide degradation. Hepatic IDE is also detected in peroxisomes where it could be involved in degrading insulin and oxidized proteins [82,83,84,85].

Although IDE plays a pivotal role in hepatic insulin clearance, recent studies have called into question its primary proteolytic function. These studies have instead emphasized its role in maintaining insulin sensitivity and glycemic homeostasis. The portal vein delivers approximately 75% of hepatic blood flow, which is enriched with postprandial nutrients, including glucose, free fatty acids, amino acids, lactate, acetate and insulin itself. These nutrients regulate IDE levels and activity in hepatocytes and skeletal muscle but not as strongly in kidney [86]. This positions IDE to act as a potential key metabolic sensor in the liver, extending its role beyond proteolysis. In fact, direct evidence of its role in insulin degradation is limited and is often derived from models of fasting/refeeding or metabolic stress. However, hepatic-specific IDE ablation (L-IDE-KO) causes post-translational modifications of INSR (reduced its plasma membrane content and its insulin-stimulated activation) without changing the expression of its *Insr* gene and of that of the insulin-like growth factor receptor, *Igf1r* [87]. This suggests that IDE indirectly exerts a negative feedback loop on hepatic insulin clearance and sensitivity. In contrast, there is no direct evidence of a reciprocal regulation of IDE protein levels or activity by insulin [87]. During prolonged fasting (18 h), IDE protein levels decrease in the livers of mice fed with a high-fat diet, whereas refeeding (30 min to 3 h) restores IDE proteolytic activity by ~45%. High-fat diet itself modulates IDE in a complex manner, showing an inverse correlation between its protein levels and insulin resistance [82,88].

Postprandial hyperglycemia following fasting is potentially mediated by cIDE isoform, which suppresses gluconeogenesis and restores glycemic homeostasis [89]. In contrast, elevated free fatty acid levels (and potentially acetate) during high-fat feeding or prolonged fasting reduce IDE protein content by roughly 30%, with the redox state further influencing these dynamics [88]. Meanwhile, portal lactate, a by-product of intestinal glycolysis, activates IDE in a dose-dependent manner, likely inducing conformational changes that enhance both protein levels and enzymatic activity [88]. These dynamic interactions highlight the integrative role of IDE in hepatic insulin clearance and metabolic adaptability.

Hepatic IDE most likely modulates insulin action through mechanisms independent of its canonical insulin-degrading function. In liver-specific gain-of-function models, IDE overexpression enhances insulin sensitivity and glucose tolerance without affecting insulin clearance or circulating hormone levels. Paradoxically, L-IDE-KO mice are protected against high-fat diet-induced glucose intolerance and insulin resistance due to preserved hepatic INSR levels. This provides evidence that IDE regulates insulin signaling via non-proteolytic roles, such as maintaining INSR and GLUT2 expression [87]. Taken together, these data support IDE’s broader regulation of insulin signaling beyond insulin clearance.

Conversely and in support of its direct role in regulating insulin degradation, *IDE* single nucleotide polymorphisms were detected in a Portuguese cohort in association with dysregulated postprandial insulin clearance in normoglycemic men to a higher extent than women [90]. Moreover, IDE expression is lower in patients with T2D [91], while its activity, but not its level (or that of CEACAM1), is reduced in African Americans in whom impairment of insulin clearance contributes to hyperinsulinemia [18]. Consistent with these clinical data, another liver-specific IDE mutant (LS-IDE-KO) that was generated by the Macedo laboratory manifests impaired glucose tolerance even on a normal chow diet, reflecting defective postprandial hepatic glucose disposal secondary to diminished insulin clearance [90]. Moreover, this mouse exhibits heightened susceptibility to diet-induced glucose intolerance and hepatic steatosis, driven by reduced GLUT2-mediated glucose uptake and upregulated CD36-mediated fatty acid transport into hepatocytes. Notably, postprandial—but not fasting—insulin clearance is profoundly reduced, promoting hyperinsulinemia that amplifies dysmetabolic vulnerability without altering systemic insulin secretion [90]. These findings demonstrate that hepatic IDE controls postprandial insulin clearance, and its altered function constitutes an early driver of dysmetabolic traits independent of changes in insulin secretion.

The conflicting findings between our laboratory [87] and those of Macedo [90] could be attributed at least partly to strain differences (C57BL/6J versus C57BL/6N, respectively). They could also highlight potential compensatory renal clearance that might mask hepatic IDE loss under fasting conditions but not postprandially. The dominant role that IDE plays in renal insulin clearance is explored below.

Beyond IDE, other proteases including lysozymes, protein disulfide isomerase (PDI), and cathepsin D contribute to insulin degradation, thereby providing functional redundancy [8,30,31,32], which may explain discrepancies in the phenotype of liver-specific IDE knockout models. Lysozymes exhibit insulin-degrading activity in lysosomal compartments, while PDI facilitates insulin processing through disulfide bond rearrangement during cellular trafficking. Cathepsin D, an aspartyl protease abundant in endo-lysosomes, cleaves insulin at multiple sites, supporting IDE-independent clearance pathways particularly under metabolic stress when hepatic IDE is compromised. This possibility deserves further investigation.

Therapeutic approaches targeting IDE in preclinical models do not yield conclusive data concerning the primary role of IDE in regulating insulin clearance versus insulin sensitivity. Whereas some IDE inhibitors cause glucose intolerance despite modest improvement of insulin sensitivity, others like 6bK, enhance glucose tolerance and insulin sensitivity likely via increasing insulin bioavailability. In contrast, sPIF, an IDE activator, ameliorates hyperglycemia at least partly by promoting insulin secretion without significantly affecting insulin clearance. Given the ability of IDE to contribute to the maintenance of glucose homeostasis by coordinating insulin and glucagon secretion [92,93], these findings underscore the distinct cellular and tissue compartmentalization of IDE’s role in regulating hepatic insulin response versus insulin secretion and degradation [8,94].

## 3. Renal Insulin Clearance

The proximal tubule cells of the kidney are the main sites of clearance of the endogenous insulin that evades hepatic extraction [95]. In contrast, they constitute the primary sites for clearing injected insulin, which bypasses portal circulation [6,24,25].

**Figure 2:**
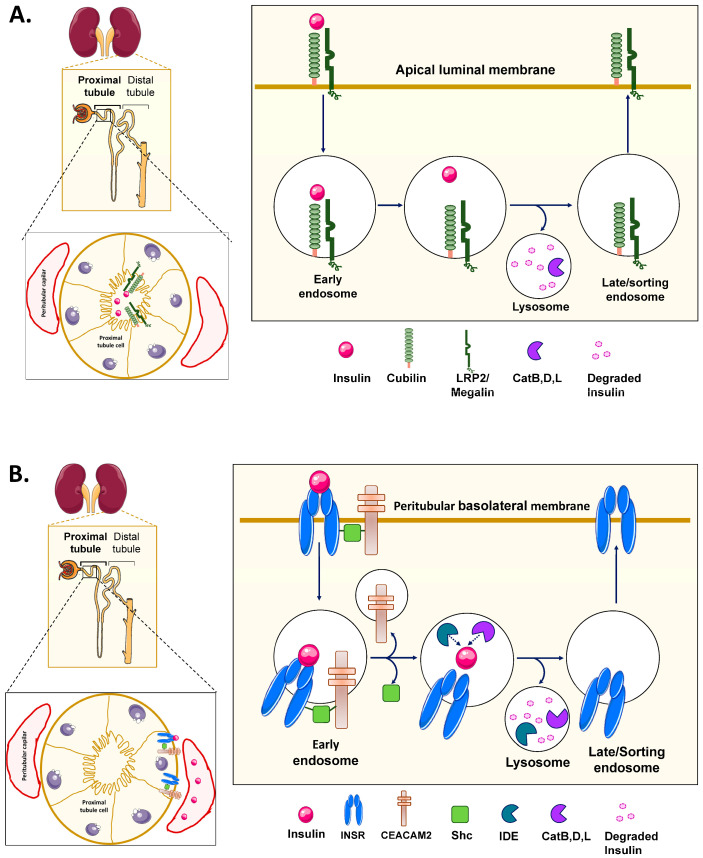
Schematic diagrams summarizing insulin uptake and degradation in proximal tubule cells of the kidney. (**A**) Approximately 60% of filtered insulin is endocytosed mainly at the apical luminal membrane via the Megalin/Cubilin receptor complex. (**B**) The remaining 40% of filtered insulin is endocytosed via INSR at the peritubular basolateral membrane. Like CEACAM1 in hepatocytes, CEACAM2 enhances the rate of receptor-mediated insulin uptake by taking part of the insulin-INSR complex. Insulin-degrading enzyme (IDE) plays a major role in insulin degradation.

Following glomerular filtration, insulin reaches proximal tubule cells via two complementary pathways, which together account for around 30–40% of systemic insulin clearance. The dominant mechanism, physiologically responsible for around 60% of renal disposal, involves non-specific reabsorption at the luminal brush border membrane via the Megalin/Cubilin receptor complex (Figure 2A). Freely filtered insulin is then rapidly endocytosed through clathrin-coated pits and undergoes degradation in the acidic milieu of lysosomes—a saturable process that shows priming effects with repeated insulin exposure and sensitivity to amino acid competition [24,25,96,97,98].

The remaining ~40% of renal clearance occurs through basolateral INSR-mediated uptake from post-glomerular peritubular capillaries to complement the apical pathway (Figure 2B). In this process, binding of insulin to INSR on the basolateral membrane triggers phosphorylation cascades and clathrin-mediated endocytosis that direct the hormone to endosomal/lysosomal degradation. This pathway maintains a constant total renal extraction rate despite fluctuating glomerular filtration rates, and it exhibits compensatory upregulation when renal filtration is reduced, as observed in chronic kidney disease [98].

Rabkin et al. [96] elegantly demonstrated that renal disease impairs both mechanisms: disrupted glomerular filtration reduces apical reabsorption, while tubular damage compromises basolateral extraction. This results in systemic insulin levels being approximately two-fold higher [96]. This dual-pathway redundancy mirrors hepatic compensation between IDE and cathepsin D, explaining why single-pathway interventions yield inconsistent outcomes.

INSR blockade with S961 in rats confirms rapid receptor-mediated insulin extraction by kidney, liver, and skeletal muscle within the first 5 min of plasma exposure, establishing these tissues as primary clearance sites [99]. High-fat feeding further demonstrates dynamic organ compensation: while hepatic clearance modestly decreases [62], renal insulin extraction increases by >50%, effectively counterbalancing impaired liver disposal to limit systemic hyperinsulinemia [99].

In obese individuals, hepatic insulin clearance declines due to insulin resistance and elevated portal free fatty acids (in part by repressing hepatic CEACAM1 transcription [51,100]), while renal insulin disposal paradoxically increases [51,101,102,103]. In the early phase of obesity, glomerular hyperfiltration and renal vasodilation stimulate glomerular filtration rate (GFR) and renal plasma flow to mediate compensatory increase in renal insulin clearance [25,97,104,105,106,107]. As obesity persists, lipotoxicity develops and receptor-mediated insulin clearance worsens, contributing to chronic hyperinsulinemia and systemic insulin resistance [108], and ultimately, kidney dysfunction [109]. Accordingly, hyperglycemic–euglycemic clamp analysis demonstrated that whole-body insulin clearance correlates more strongly with lean mass and insulin sensitivity than with effective renal plasma flow or GFR in adult patients with T2D and normal kidney function [25]. This underscores the important role of receptor-mediated insulin uptake at the basolateral domain of kidney proximal tubules relative to filtered and reabsorbed insulin at the apical region [99]. It also proposes a previously underestimated role for insulin sensitive tissues (muscle, adipose, brain) in systemic insulin clearance in this population [25].

The prevalence of chronic kidney disease (CKD) is on the rise particularly in adults at ≥60 years of age [110]. Consistent with hyperinsulinemia contributing strongly to age-related metabolic alterations [1], cross-sectional and clinical studies have identified insulin resistance and hyperinsulinemia as potential independent risk factors in the absence of diabetes in patients with CKD [111,112,113], in addition to inflammation and oxidative stress [114]. In these patients and in patients with renal failure, renal insulin clearance is diminished. This prolongs the life of insulin [96], necessitating a lower dose of injected insulin to avoid hypoglycemia [115]. Therefore, it is imperative to delineate the basic mechanisms of renal insulin clearance and whether alteration in this process contributes to systemic hyperinsulinemia in CKD.

### 3.1. Role of CEACAM1 in Insulin Uptake in Proximal Tubule Cells of the Kidney

The dominant and most abundant CEACAM isoform in murine kidney is CEACAM2. The intracellular domains of CEACAM2 and CEACAM1 are highly homologous (~95%) [116,117]. The most dominant form of CEACAM2 in the kidney (CEACAM2-2L) contains two instead of four IgG-like structures in its extracellular domain (hereafter refers to as CEACAM2 for simplicity). In contrast to CEACAM1, which is highly conserved, CEACAM2 is only expressed in mice (murine *Ceacam1* and *Ceacam2* genes are the orthologs of human *CEACAM1* gene).

CEACAM2 is restricted to proximal tubule cells, the main site of renal insulin clearance. Like CEACAM1, CEACAM2 undergoes phosphorylation on the conserved YTVL tyrosine phosphorylation site [118]. Furthermore, [^125^I]-insulin uptake revealed reduced receptor-mediated insulin uptake in proximal tubule cells isolated from *Ceacam2* null (*Cc2*^−/−^) relative to wild-type mice [118]. This proposes that CEACAM2-dependent mechanisms underlie receptor-mediated insulin uptake in kidney proximal tubule cells.

Despite altered insulin uptake in renal proximal tubule cells of *Cc2*^−/−^ mice, the metabolically active *Cc2*^−/−^ male mice do not develop chronic hyperinsulinemia and insulin resistance until about 9–10 months of age [119]. At this age, hepatic insulin clearance becomes impaired owing to the >60% loss in hepatic CEACAM1 expression [120]. This aligns with the dominant role of hepatic insulin clearance in modulating insulin response. Similarly, mirroring the link between chronic hyperinsulinemia, insulin resistance, and impaired kidney function, *Cc2*^−/−^ mice exhibit spontaneous tubular injury that progresses to proteinuria, reduced glomerular filtration rate, and overt renal dysfunction [118]. Further studies are needed to dissect out the role of impaired CEACAM-dependent renal insulin clearance in kidney dysfunction.

### 3.2. Role of LRP2 (Megalin) in Insulin Uptake in Proximal Tubule Cells of the Kidney

Whereas INSR is the main receptor-mediating insulin signaling in hepatocytes and LRP1 contributes to hepatic insulin sensitivity and receptor trafficking, LRP2 (Megalin) mediates proximal tubular uptake of glomerularly-filtered insulin.

LRP2/Megalin, is a ~600 kDa type I membrane glycoprotein receptor that, unlike LRP1, is not cleaved and it remains as a single polypeptide [121,122]. LRP2 continuously recycles between the apical luminal membrane and the subapical endosomes of proximal tubule cells [69,123]. This apical–endosomal trafficking is tightly regulated by the NPXY motifs in its cytoplasmic tail. As in the constitutively recycling LDL receptor [124], NPXY recruits endocytic adaptors such as Disabled-2 (Dab2) and clathrin/AP-2. Following internalization, the endocytosis complex is targeted to endosomes, where Rab GTPases coordinate its endosomal sorting and recycling. Hormones (like estrogen) and metabolic cues (fatty acid activators of PPARα/γ) modulate renal LRP2 expression and phosphorylation, thereby influencing its endocytotic function [122,123,125,126,127,128].

LRP2/Megalin acts in partnership with Cubilin to mediate receptor-dependent insulin endocytosis and its targeting to endosomes for lysosomal degradation, a process that prevents urinary loss of insulin [129,130,131]. Consistently, *Lrp2*-deficient mice exhibit glucosuria, elevated urinary insulin and peptide excretion, and hyperinsulinemia, consistent with defective insulin reabsorption [123]. In rats, Megalin expression is reduced in kidney disease associated with early-stage diabetes [132,133] and aging [134]. In humans, similar alterations are observed in renal disorders, such as nephrosis and Donnai–Barrow syndrome [128,135,136]. Genetic alterations in LRP2/Megalin and Cubilin genes have been associated with insulin resistance and proteinuria in patients with T2D and end-stage renal disease in populations with recent African ancestry [137].

Together with hyperinsulinemia, patients with T2D and metabolic syndrome exhibit elevated circulating levels of angiotensin II and leptin among other potentially nephrotoxic molecules. Once filtered by the glomerulus, these macromolecules are transported to the proximal tubule cells by LRP2/Megalin. Their sustained uptake progressively overloads the intracellular metabolic and lysosomal degradation pathways, a process that promotes cellular hypertrophy and oxidative stress [130,138,139,140]. Oxidative stress, hyperglycemia and glucosuria cause a compensatory increase in LRP2/Megalin expression in early-stage diabetes to handle protein overload and prevent proteinuria. As proximal tubular damage progresses with sustained hyperglycemia in diabetes, proteinuria develops. This suggests that therapeutic modulation of LRP2/Megalin and its downstream signaling pathways (PI3K/Akt/mTORC1) could reduce tubular endocytic load and subsequently attenuate renal injury in diabetes [130,138,139,140,141,142].

To summarize, growing evidence shows that LRP2/Megalin functions as a central player in epithelial endocytic networks, coordinating the uptake of filtered hormones, nutrients, and vitamins with insulin metabolism. Thus, altered LRP2/Megalin function links renal tubular defects with metabolic dysregulation. This emphasizes the regulatory role of LRP2/Megalin in renal protein absorption and insulin metabolism, and subsequently, in preventing proteinuria and hyperinsulinemia in renal failure associated with T2D [20,24,131].

### 3.3. Role of IDE in Renal Insulin Clearance

Despite ongoing debate, the current literature suggests that IDE’s proteolytic function predominates physiologically in renal insulin degradation relative to other proteases and its action is more important in renal insulin clearance than in liver and skeletal muscle [88]. This concept is reinforced by high IDE expression and by its identification as the most abundant proteolytic enzyme in proximal tubule cells. Furthermore, the distribution of IDE to the brush border membrane, plasma membrane, perinuclear region and cortical endosomal compartments positions it to participate in both filtered and INSR-mediated internalized insulin [14,82,143].

IDE initiates the proteolysis of endosomal receptor-bound insulin by cleaving the disulfide bonds of its B-chain while leaving those of A-chain intact. This is followed by the reduction of these bonds by protein disulfide isomerase (PDI) for further lysosomal completion. Supporting the role of IDE in renal insulin handling, inhibiting its enzymatic activity by bacitracin increases the release of intact insulin [144].

Regulation of IDE is consistent with its role in renal insulin degradation. For instance, insulin interacts with IDE to enhance its interaction with Sorting Nexin 5 (SNX5) [144]. IDE-SNX5 interaction upregulates IDE expression via PI3K/Akt signaling and directs it to the plasma membrane and perinuclear sites in proximal tubule cells [143]. In contrast, SNX5 depletion represses IDE mRNA and protein levels and its enzymatic activity to impair renal insulin clearance [145]. In partial support of the role of IDE-SNX5 interaction in renal insulin clearance, renal IDE and SNX5 levels are reduced in parallel to elevated serum insulin in spontaneously hypertensive rats (SHRs) and in renal proximal tubule cells from hypertensive human subjects [145].

High-fat diet inhibits PPARγ binding to the *Ide* promoter to suppress its transcription [143]. This impairs insulin degradation in proximal tubule endosomes and membrane to contribute to systemic hyperinsulinemia [143]. In contrast, rosiglitazone treatment restores IDE expression and enhances insulin clearance [143]. Together, these findings highlight the pivotal role of IDE in renal tubular insulin degradation [143,144].

## 4. Concluding Remarks and Future Directions

Despite decades of research, the mechanisms underlying insulin clearance along the liver–kidney axis have not been fully elucidated. Whereas the role of CEACAM proteins in receptor-mediated insulin uptake in hepatocytes has been widely characterized, its role in receptor-mediated insulin uptake at the peritubular basolateral membrane of proximal tubule cells is emerging. At the apical luminal membrane of these cells, Megalin/Cubilin- mediated insulin uptake dominates.

Moreover, the proteases that are primarily responsible for insulin degradation have not been fully identified. Overall, endosomal degradation appears to dominate in liver whereas lysosomal degradation occurs more favorably in kidney. Whereas IDE has traditionally been considered as the dominant enzyme in insulin degradation, emerging evidence suggests that a network of redundant proteases, including lysosomes, cathepsin D and PDI, are involved. Classical proteolytic approaches, including IDE-targeted inhibitors (e.g., bacitracin and 6bK), have produced inconsistent results across models. Cathepsin D inhibitors (pepstatin analogues) and lysosomal modulators (chloroquine derivatives) targeting redundant degradation pathways are particularly relevant when IDE is saturated postprandially. Thus, comparative proteomics and functional rescue experiments are essential to more effectively identify cell-specific proteases that are responsible for insulin degradation along the liver–kidney axis.

**Figure 3:**
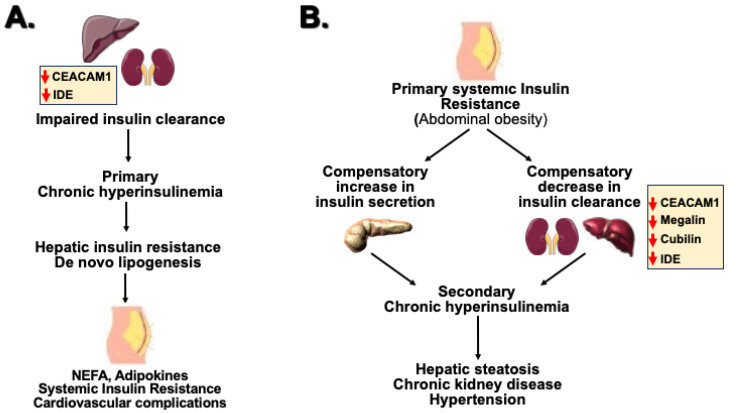
Schematic diagrams of the chicken-and-egg relationship between hyperinsulinemia and insulin resistance. (**A**) Hyperinsulinemia driven by impaired insulin clearance along the liver–kidney axis can cause systemic insulin resistance and ultimately abdominal obesity with cardiometabolic abnormalities. (**B**) Systemic insulin resistance commonly associated with abdominal obesity causes secondary hyperinsulinemia (driven by increased insulin secretion and altered insulin clearance) as a compensatory mechanism. Red downward arrows denote a decrease in the expression level of indicated proteins.

In summary, this review presents current CEACAM1-based evidence that impaired insulin clearance along the liver–kidney axis can play a causal role in systemic insulin clearance (Figure 3A). This paradigm challenges the classical view that impaired insulin clearance is merely a compensatory mechanism to counter systemic insulin resistance that is often associated with abdominal obesity (Figure 3B).

The mechanisms described above predict that upstream interventions that target the rate-limiting steps of insulin capture and internalization could bypass protease redundancy with greater tissue specificity. For instance, CEACAM1 upregulators (such as GLP-1R agonists) could amplify receptor-mediated insulin internalization along the liver–kidney axis to clear excess insulin and maintain its physiologic levels [146]. Megalin/Cubilin modulators offer the prospect of specifically enhancing insulin clearance in kidney and its function without interfering with the liver.

The therapeutic efficacy may vary by the stage of diabetes depending on whether clearance defects precede or follow insulin resistance. For instance, it is reasonable to predict that patients with early insulin resistance may benefit from promoters of insulin clearance to limit chronic hyperinsulinemia and prevent glucose dysregulation and systemic insulin resistance. Ultimately, precision pharmacology requires the use of biomarkers to distinguish between primary and secondary clearance defects. Once causal relationships are clarified, these approaches position insulin clearance along the liver–kidney axis as a viable therapeutic target of insulin resistance.

## Data Availability

No new data were created or analyzed in this study. Data sharing is not applicable to this article.

## Supplementary Materials

The following supporting information can be downloaded at: https://www.mdpi.com/article/10.3390/metabo16070439/s1, Table S1: Genetically-modified Mouse Models to study hpatic and renal rnsulin clearance.

## Abbreviations

The following abbreviations are used in this manuscript:

T2D	Type 2 Diabetes
CEACAM1	Carcinoembryonic Antigen-Related Cell Adhesion Molecule 1
IDE	Insulin-Degrading Enzyme
LRP	Low-Density Lipoprotein Receptor-Related Protein
