# Supplementary material for: Insulin Clearance Along the Liver–Kidney Axis: Implications for Insulin Action
Source: Metabolites. 2026 Jun 24;16(7):439. doi: 10.3390/metabo16070439 (PMC13413472; doi:10.3390/metabo16070439)
Supplement: Supplementary file 1 [file metabolites-16-00439-s001.zip › metabolites-4301771-supplementary.pdf]

Genetically-modified Mouse Models to study hpatic and renal rnsulin clearance

Model	Type of Modification	Effect on Insulin Clearance	Metabolic and Renal Phenotype	Reference
L-SACC1	Transgenic mouse with liver-specific dominant-negative inactivation of Ceacam1: Liver-specific overexpression of the non-phosphorylatable Ser503 to Alaine. CEACAM1 mutant	↓↓ Hepatic insulin clearance	Chronic hyperinsulinemia at 2 months of age, followed by systemic insulin resistance, steatohepatitis and visceral obesity. Mice also exhibit hepatic fibrosis	PMID: 11850617<br>PMID: 16638824<br>PMID: 18848945<br>PMID: 15316023
Cc1$^{-/-}$ null	Global ablation of *Ceacam1* gene	↓↓ Hepatic insulin clearance	Chronic hyperinsulinemia at 2 months of age, followed by glucose intolerance, insulin resistance, steatohepatitis, and hepatic fibrosis. Mice also exhibit atherosclerosis, hypertension and endothelial dysfunction	PMID: 2518480<br>PMID: 23734002<br>PMID: 23800882

Cc1$^{-/-\ liver+}$	*Cc1*$^{-/-}$ mouse with exclusive liver-specific transgenic reconstitution of Ceacam1.	Insulin clearance is fully restored	Normo-insulinemia' Restored insulin sensitivity and metabolic function, reversed hepatic injury and restored endothelial and cardiac function	PMID: 28913615<br>PMID: 29396368<br>PMID: 34058224<br>PMID: 28913658
**Liver-specific Ceacam1 KO**	Liver-specific KO (*AlbCre+Cc1*$^{fl/fl}$)	↓↓ Hepatic insulin clearance	chronic hyperinsulinemia at 2 months of age, followed by secondary hepatic insulin resistance and ssteatohepatitis at 5-6 months of age, followed by visceral obesity and systemic insulin resistance at 9 months of age.<br><br>Mice also exhibited hepatic fibrosis and atherosclerosis	PMID: 30664851<br>PMID: 38381498<br>PMID: 33163831

**CEACAM2 Global KO (Cc2-/-)**	Global ablation of Ceacam2 gene.	↓↓ Renal insulin clearance + progressive decline in hepatic clearance	Sex dimorphism with males exhibiting chronic hyperinsulinemia and insulin resistance at 10 months of age and females at 2 months of age. Mice develp an increase in GLP1-mediated insulin secretion, ,progressive glucose intolerance, progressive chronic kidney disease	PMID: 41509249<br>PMID: 22159884<br>PMID: 20381490<br>PMID: 26586918<br>PMID: 28567513<br>PMID: 22159884
**Global IDE KO**	Global ablation of *Insulin-Degrading Enzyme (Ide) gene*	↓ Systemic insulin clearance	↑ Fasting plasma insulin, ↑ glucose, glucose intolerance, testicular and sperm abnormalities	PMID: 31198829
**L-IDE-KO** (Liver-specific IDE KO on C57BL/6J)	Liver-specific KO of *Ide* gene	Clearance similar to control	Glucose intolerance, insulin resistance	PMID: 30098324<br>PMID: 32916153

**LS-IDE-KO** (Liver-specific IDE KO on C57BL/6N)	Liver-specific KO of I*de* gene	↓ Clearance similar to control in fasting (normal diet), but ↓ **postprandial** insulin clearance	Glucose intolerance, hepatic steatosis with high-fat diet, postprandial insulin resistance	PMID: 33631143
**Silencing SNX5** (*Sorting Nexin 5*)	↓ IDE expression and function	↓ insulin clearance	Hyperinsulinemia, hyperglycemia, systemic insulin resistance	PMID: 29080077
**Adenoviral IDE overexpression in liver**	Adenoviral-mediated overexpression of IDE in liver	Normal Clearance	↓ Insulinemia, ↑ insulin sensitivity, ↓ blood glucose, improved insulin resistance	PMID: 32916153

**Megalin (LRP2) KO**	Global ablation of LRP2/megalin gene	Filtered insulin is not degraded in the kidneys and is excreted directly in the urine (insulinuria). Metabolic renal clearance (breakdown) decreases, but the net elimination of the hormone from the body shifts toward urinary excretion.<br><br>Intact hepatic clearance<br><br>↓SGLT2	Reduced glucose reabsorption, leading to basal glucosuria and maintaining glucose levels within normal range. However, when mice are fed a Western diet, the renal tissue loses its adaptive capacity, which drastically alters glucose handling and triggers a marked diet-induced glucose intolerance.<br><br>Absence of severe basal hyperinsulinemia<br><br>do not develop insulin resistance primarily due to the absence of chronic systemic hyperinsulinemia in the early stages. However, the	PMID: 38984983

			combination of megalin loss with the lipotoxicity of the Western diet selectively sensitizes the kidney, causing structural damage (tubular injury and fibrosis). It is this chronic tissue damage that ultimately promotes an inflammatory environment conducive to localized insulin resistance.	
**Cubilin (CUBAM) KO**	Global deletion of cubilin gene	↓ Renal insulin clearance and is excreted directly in the urine (insulinuria). (interacts with megalin)	Absence of severe basal hyperinsulinemia.<br><br>Normal baseline tolerance, but vulnerable under metabolic stress (HFD).<br><br>Absence of primary peripheral resistance	PMID: 34747197

**Megalin/Cubilin Double KO**	Combined global deletion of both receptors	A sharp decline in metabolic renal clearance and massive insulinuria	Absence of systemic basal hyperinsulinemia.<br><br>Improved or normal glucose tolerance at baseline;<br><br>Absence of primary peripheral insulin resistance.	PMID: 21926402
